# Clinical and Anatomical Factors Affecting Recurrent Laryngeal Nerve Paralysis During Thyroidectomy *via* Intraoperative Nerve Monitorization

**DOI:** 10.3389/fsurg.2022.867948

**Published:** 2022-04-28

**Authors:** Nurcihan Aygun, Mehmet Kostek, Mehmet Taner Unlu, Adnan Isgor, Mehmet Uludag

**Affiliations:** ^1^Department of General Surgery, Sisli Hamidiye Etfal Training and Research Hospital, Istanbul, Turkey; ^2^Department of General Surgery, Sisli Memorial Hospital, Istanbul, Turkey

**Keywords:** thyroidectomy, RLN injury, intraoperative neuromonitoring (IONM), RLN branching, inferior thyroid artery (ITA)

## Abstract

**Background:**

Despite all the technical developments in thyroidectomy and the use of intraoperative nerve monitorization (IONM), recurrent laryngeal nerve (RLN) paralysis may still occur. We aimed to evaluate the effects of anatomical variations, clinical features, and intervention type on RLN paralysis.

**Method:**

The RLNs identified till the laryngeal entry point, between January 2016 and September 2021 were included in the study. The effects of RLN anatomical features considering the International RLN Anatomical Classification System, intervention and monitoring types on RLN paralysis were evaluated.

**Results:**

A total of 1,412 neck sides of 871 patients (672 F, 199 M) with a mean age of 49.17 + 13.42 years (range, 18–99) were evaluated. Eighty-three nerves (5.9%) including 78 nerves with transient (5.5%) and 5 (0.4%) with permanent vocal cord paralysis (VCP) were detected. The factors that may increase the risk of VCP were evaluated with binary logistic regression analysis. While the secondary thyroidectomy (OR: 2.809, 95%CI: 1.302–6.061, *p* = 0.008) and Berry entrapment of RLN (OR: 2.347, 95%CI: 1.425–3.876, *p* = 0.001) were detected as the independent risk factors for total VCP, the use of intermittent-IONM (OR: 2.217, 95% CI: 1.299–3.788, 0.004), secondary thyroidectomy (OR: 3.257, 95%CI: 1.340–7.937, *p* = 0.009), and nerve branching (OR: 1.739, 95%CI: 1.049–2.882, *p* = 0.032) were detected as independent risk factors for transient VCP.

**Conclusion:**

Preference of continuous-IONM particularly in secondary thyroidectomies would reduce the risk of VCP. Anatomical variations of the RLN cannot be predicted preoperatively. Revealing anatomical features with careful dissection may contribute to risk reduction by minimizing actions causing traction trauma or compression on the nerve.

## Introduction

Recurrent laryngeal nerve (RLN) is one of the most important anatomical structures at risk during thyroidectomy. In literature, it has been reported that factors such as thyroid cancer, neck dissection, Graves' disease, thyroiditis, large goiter, retrosternal goiter, recurrent benign, and malignant diseases, complete resection of the thyroid lobe, uncertain identification of RLN, reoperation for postoperative bleeding, previous neck radiotherapy, anatomic variations, low and medium hospital volume, low surgeon volume, extralaryngeal branching nerve, non-recurrent laryngeal nerve were related with an increased risk of post-thyroidectomy RLN paralysis (1–8).

Routine dissection and visual identification of the RLN were defined by Lahey in 1938 and are still the gold standard method (9).

It could be difficult to visually identify the RLN intraoperatively due to the factors such as anatomical variations of the RLN, recurrent goiter, large substernal goiter, and locally advanced thyroid cancer, which may be related to the increased risk of RLN paralysis (8).

Although many clinical factors associated with an increased risk of RLN paralysis can be predicted preoperatively, especially anatomical variations of the RLN cannot be predicted preoperatively (6).

With the improving data regarding the anatomy and function of the RLN, the use of intraoperative nerve monitoring (IONM), one of the technical and technological developments allowing functional evaluation of the nerve in addition to visual identification has been increasing recently (10, 11).

The visual identification rate of the nerve is increased by IONM, allowing the early localization and identification of RLN (8). Whether IONM reduces the risk of RLN paralysis is still controversial, and there are still studies reporting that it has no significant effect on RLN paralysis (12).

It has been demonstrated that the use of IONM reduces the risk of RLN injury (13), and continuous IONM (CIONM) is superior to intermittent IONM (IIONM) in preventing RLN injury (11, 14).

In many studies anatomical variations of RLN and its relationship with various landmarks such as Zuckerkandl tubercle, inferior thyroid artery (ITA), and Berry ligament have been investigated (15–20).

The International RLN anatomical classification system was published in 2016, including the trajectory of the main trunk of the RLN and its potential clinically important features such as extralaryngeal branching, neural entrapment, invasion, diameter of the nerve, dynamic components of surgery related to postoperative glottic function such as signal loss and extensive neural dissection. The estimated rates for the prevalence of anatomical features and RLN trajectory includes a literature review and expert opinions of the International Neural Monitoring Study Group (INMSG) (21).

A prospective international multicentric study evaluating 1,000 nerves considering this classification system and its associated RLN paralysis was recently published (22).

The study group continues to collect data and is preparing to publish the second part of the study which includes 5,000 nerves. The number of studies evaluating anatomical factors affecting RLN injury is limited. In this study, we aimed to evaluate the risk factors for RLN paralysis, including the anatomical data according to the International RLN Anatomic Classification System.

## Materials and Methods

### Study Population

The data of patients who underwent thyroidectomy (± parathyroidectomy) with IONM between January 2016 and August 2021 were evaluated retrospectively.

Demographic, clinical, anatomical, IONM, and operative data of all patients have been recorded in the clinical standard database in detail and informed consents have been obtained from the patients for data collection.

Approval was obtained from the local ethics committee and patients' data were analyzed according to the guidelines in the Helsinki Declaration.

**Figure 1:**
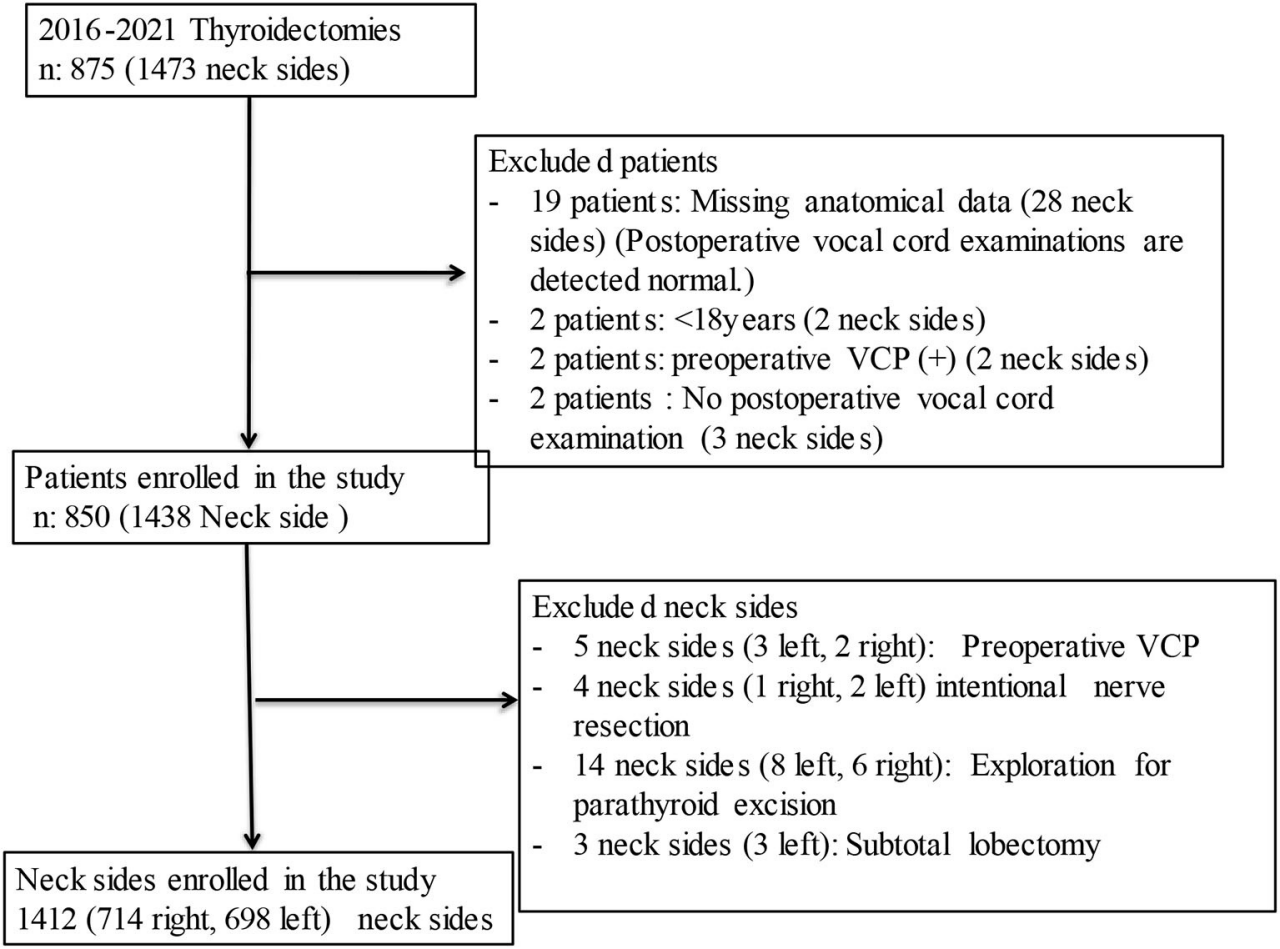
Flow diagram of the patients enrolled in the study.

In the analysis of the data, each nerve at risk on each operated neck side was considered as a separate entity. Patients with preoperative vocal cord paralysis (VCP), intentional resection of the RLN due to tumor invasion, neck side where only parathyroidectomy or subtotal thyroidectomy was performed, neck sides without fully explored RLNs, neck side with missing data of RLN's anatomical features, and patients without postoperative vocal cord examinations, patients younger than 18 years of age were excluded from the study (Figure 1).

Patient's gender, age, body mass index, type of surgery performed (primary or secondary intervention), the extent of surgery (thyroidectomy with or without central neck dissection), type of neuromonitoring, presence of preoperative hyperthyroidism, postoperative diagnosis, pre- and postoperative vocal cord examinations, side of the nerve on the neck, anatomical trajectory of the RLN in the neck according to International RLN anatomical classification and anatomically important neural features, the relationship of RLN and inferior thyroid artery, the Zuckerkandl tubercle (ZT) grade, and weight of the removed thyroid lobe were evaluated.

It was defined as extralaryngeal branching in case of RLN branching at least >5 mm before its laryngeal entry point and all its branches entering the larynx (23). Zuckerkandl tubercle (ZT) size was classified into 4 degrees by Pelizzo et al. If it is not seen it is graded as 0 degree, only thickening on the lateral edge of the thyroid lobe is graded as 1st degree, <1 cm is graded as 2nd degree, >1 cm is graded as 3rd degree. ZT 0 and 1st degrees were accepted as a category, 2nd and 3rd degrees were accepted as another category (24).

According to the international RLN anatomical classification system, the anatomical trajectory of the RLN in the neck is evaluated as normal course, acquired, or embryologic variation. Left normal trace (L1); RLN running parallel to the tracheoesophageal groove or at an angle of less than 30 degrees, right normal trace (R1); RLN running at an angle of 15–45 degrees relative to the tracheoesophageal groove, acquired variations; lateral displacement of the nerve on the left (L2a), medial displacement (R2a) on the right, ventral displacement on the right and left (R2b, L2b, respectively), embryological variation was defined as non-recurrent nerve (R3 on the right, L3 on the left).

Clinically important features were evaluated whether fixed/splayed/entrapped (F), presence of nerve invasion (I), and RLN entrapped by Berry ligament posterior fibers or vascular structures (L) (21). All of the invaded nerves were excluded from the study because they had preoperative vocal cord paralysis or were resected intentionally.

### IONM and Thyroidectomy

All surgeries were performed under general anesthesia. All patients were intubated with a surface electrode-based endotracheal tube and a low-dose neuromuscular agent (recuronium 0.3 mg/kg) for induction. Muscle relaxants were not administered after induction. NIM 3.0 Nerve Monitoring system (Medtronic xomed, Jacksonville) with endotracheal tube was used. IONM was applied intermittently or continuously. Factors such as the choice of the surgeon depending on the case and the availability of the Automatic Periodic Stimulation (APS) probe (Medtronic Xomed Inc. Jacksonville, FL, USA) for the patient were effective in the selection of the IONM method. IONM installment, anesthesia induction and maintenance, tube position verification tests, IONM application (4-step procedure: V1, R1, R2, V2), and data evaluation were assessed according to the International nerve monitoring study group guidelines (25).

For intermittent IONM (IIONM), a monopolar stimulator probe (Medtronic Xomed) was used. The stimulations were applied with a current intensity of 1 mA, the stimulation duration of 100 ms, the threshold value of 100 mV, and the current frequency of 4 Hz.

Medtronic APS probe was used for continuous IONM (CIONM). At the level of the thyroid mid pole, the carotid sheath was opened, the vagus was dissected 360 degrees, and the APS probe was applied to the vagus and the electrode was connected to the monitor system. Baseline latency and amplitude responses were calibrated by giving 20 automatic periodic stimulations to the vagus nerve from the monitoring system. The system is set to stimulate with a current of 1 mA every second. Although the expected initial baseline value for CIONM was 500 μV, CIONM was applied at values above 300 μV. The device was set to give an audible and visual warning when the amplitude value is 50% below the baseline and/or the latency value increases by more than 10% of the baseline value.

The signal loss was defined as a decrease in amplitude below 100 μV or failure to receive it with supramaximal stimulation (25).

### Operational Technique

The approach to the thyroid gland was through the midline with an anterior approach between the strap muscles on both sides in primary thyroidectomies and/or in patients undergoing central neck dissection.

In secondary thyroidectomies and/or central dissection, the approach to the thyroid gland was through between the strap muscles and the sternocleidomastoid muscle with a backdoor lateral approach.

The sternohyoid and sternothyroid (or just the sternothyroid muscle) were divided 1/3 cranially to increase exposure and perform safer dissection in patients with large goiters and/or substernal goiters and patients with short necks.

In secondary central dissections, the sternothyroid muscle was routinely divided. After reaching the thyroid gland, the vagus nerve was found in the carotid sheath firstly and stimulated by the probe and the V1 response was recorded.

In general, dissection of the thyroid lobe was initiated from the upper pole. IONM was used for the identification, verification, and mapping of the external branch of the superior laryngeal nerve (EBSLN) during the dissection of the superior thyroid vessels.

In primary surgery, RLN was usually identified at the level of the inferior thyroid artery (ITA). The thyroid gland was carefully dissected from the anteromedial aspect of the RLN up to its entrance to the larynx. If the nerve was branched at the proximal of ITA, it was dissected to the point where it branched proximally.

In secondary interventions, to avoid scar tissue, the RLN was identified with an inferior approach, and it was mapped till its laryngeal entry point.

In severely large goiters or goiters with a large substernal component, the RLN was identified at the laryngeal entry point with a superior approach and dissected proximally. Rarely, in substernal or retropharyngeal enlarged goiters, the RLN was identified in the region of Berry's ligament with a medial approach. In cases with the persistent signal reduction due to the thyroid lobe traction in the lateral or superior approach, the medial approach was also preferred (26).

At the end of the operation, surgical data, vagus anatomical features, RLN anatomical trajectory and clinically important features, relationship with ITA, extralaryngeal branching features, the presence of Zuckerkandl tubercle, and its relationship with RLN, EBSLN anatomical features, IONM data were entered into the clinical database in detail.

In patients who were planned for bilateral intervention, the side where the malignancy was localized, the side with the dominant lobe or nodule was operated first. Staged thyroidectomy was performed in patients who developed signal loss on the first side, except for patients with a diagnosis of high and intermediate risk malignancy.

Completion of thyroidectomy to the contralateral lobe in patients who has a diagnosis of malignancy in the final pathology result after lobectomy or surgical intervention to the second side in staged thyroidectomy was accepted as the primary intervention.

The mechanisms of RLN injury were examined under 5 main headings; traction trauma due to medial traction and elevation of the thyroid lobe (A), blunt trauma with a surgical instrument during dissection of the nerve, contusion, accidental clamping of the nerve, pressure, compression, or suction with a surgical instrument (B), clipping or ligating during or after dissection of the nerve during the separation of small vessels or connective tissue, touching the nerve directly with electrocautery or energy-based device, or thermal injury by lateral heat spread (D), dividing the nerve due to visual misidentification (E) (22, 27).

Preoperative and postoperative vocal cord examinations were performed by an independent otolaryngologist. Postoperative periodic vocal cord examinations were applied to patients with postoperative VCP. If VCP improved in 12 months, it was defined as a transient, if it still persisted at 12 months, it was defined as permanent VCP (27).

### Statistical Analysis

Data were evaluated in the IBM SPSS Statistics version 25.0 program (IBM, Armonk, NY, USA). Data are presented as mean ± SD. The normal distribution of the data was tested and appropriate parametric or non-parametric tests were selected. Pearson Chi Square and Fisher exact tests were used to compare categorically independent groups, and the odd ratio was calculated for significant differences in 2 × 2 tables. To evaluate the independent factors affecting VCP, the formula formed from the features that were significant in pairwise comparisons was evaluated by binary logistic regression analysis. *p* < 0.05 values were considered statistically significant.

## Results

Twenty-five patients (35 neck sides) of 875 patients who were operated on during the study period were excluded from the study. Completion thyroidectomy was performed in 21 of 850 patients, and a total of 871 operations were performed. The bilateral intervention was performed in 566 of 850 patients and unilateral intervention in 306 patients, and a totally 1,438 neck side interventions were performed in 850 patients. According to the exclusion criteria, 26 neck sides were excluded. A total of 1,412 neck sides were included in the study, 1,089 (77.1%) neck sides in 657 female patients and 323 (22.9%) neck sides in 193 male patients. In total, 714 (50.1%) of the nerves at risk were on the right side and 698 (49.9%) were on the left side.

IIONM was applied in 692 (49%) RLNs and CIONM was applied in 720 (51%) RLNs. Primary intervention was performed on 1,329 (94.1%) neck sides, and secondary intervention was performed on 83 (5.9%) neck sides. Central neck dissection was performed on 147 (10.4%) neck sides. The relationship between RLN and ITA was evaluated on the 1,396 neck sides. In total, 675 (48.4%) of the RLNs were crossing anterior to ITA, 596 (42.7%) of the RLNs were crossing posterior to ITA, and 125 (8.9%) of RLNs were crossing between the ITA branches. In total, 482 (34.1%) of RLNs had extralaryngeal branches, 449 (32.8%) had 2 branches, and 33 (2.3%) had 3 and more than 3 branches. Zuckerkandl's tubercle was evaluated in 1,291 lobes, of which 788 (61%) were 0 or 1st degree, and 503 (39%) were 2nd or 3rd degree.

RLNs (88.2%) were R1/L1, 7.8% of RLNs were R2a/L2a, 3.1% of RLNs were R2b/L2b, and 0.6% of RLNs were R3 according to the International RLN anatomical classification. R3 (non-recurrent nerves) were all on the right side, the ratio was 1.1% on the right side. RLN course was evaluated in 1,124 nerves, and 950 (84.5%) nerves had no features, 43 (3.8%) nerves were fixed, 59 (5.3%) nerves were splayed, 72 (6.4%) nerves were entrapped. Three hundred and seven (23.5%) of 1,305 nerves were entrapped in the Berry region by Berry fibers and/or vascular structures.

### VCP

VCP was detected in 83 (5.9%) of the total 1,412 RLNs, of which 78 (5.5%) were transient and 5 (0.4%) were permanent.

In paired comparison, total VCP rates in the secondary intervention compared to primary intervention (13.3 vs. 5.4%, *p* = 0.005, respectively), RLNs crossing anterior to ITA, compared to those crossing posterior to ITA and between the branches of ITA (8.4 vs. 3.2 vs. 3.2%, *p* = 0.000, respectively), extralaryngeal branching nerves compared to those without branching (8.1 vs. 4.7%, *p* = 0.011, respectively), RLNs with entrapment by Berry fibers and/or vascular structures compared with those without entrapment (9.8 vs. 4.6%, *p* = 0.001, respectively) were higher. No significant difference was found in terms of other factors (Table 1).

**Table 1:** Evaluation of clinical and anatomical factors for total VCP by univariant analysis.

**Feature**		***n***	**VCP *n* (%)**	**OR (95% CI min–max)**	***p***
Age					
	No VCP	1,329 (94.1%)		49.1 ± 13.5 (18–89)	0.673
	VCP	83 (5.9%)		49.5 ± 11.7 (23–79)	
Gender		1,412			
	Female	1,089 (77.1%)	65 (6%)	0.928 (0.542–1.588)	0.785
	Male	323 (22.9%)	18 (5.6%)		
BMI		1,230			0.883
	No VCP	1,170		28.8 ± 6 (16.3–63)	
	VCP	60		28.7 ± 5.6 (18.4–44.4)	
Neck side of the nerve					
	Right	714 (50.1%)	46 (6.4%)	0.813 (0.520–1.270)	0.362
	Left	698 (49.9%)	37 (5.3%)		
Type of nerve monitoring					
	IIONM	692 (49%)	49 (7.1%)	1.538 (0.980–2.413)	0.061
	CIONM	720 (51%)	34 (4.7%)		
Type of intervention					
	Primary	1,329 (94.1%)	72 (5.4%)	0.375 (0.190–0.738)	0.005*
	Secondary	83 (5.9%)	11 (13.3%)		
Central neck dissection					
	Applied	147 (10.4%)	10 (6.8%)		0.615
	Not applied	1,265 (89.6%)	73 (5.8%)	0.839 (0.423–1.663)	
RLN-ITA relationship		1,396			0.000*
	Anterior to ITA	675 (48.4%)	57 (8.4%)	2.790 (0.994–7.833)	0.051
	Posterior to ITA	596 (42.7%)	19 (3.2%)	0.996 (0.333–2.980)	0.994
	Between branches of ITA	125 (8.9%)	4 (3.2%)		
Nerve branching		1,412			0.011*
	Non-branching	930 (65.9%)	44 (4.7%)	1.773 (1.135–2.769)	
	Branching	482 (34.1%)	39 (8.1%)		
Entrapment of RLN by Berry ligament		1,305			
	Yes	307 (23.5%)	30 (9.8%)	2.241 (1.388–3.619)	0.001*
	No	998 (76.5%)	46 (4.6%)		
Zuckerkandl tubercle		1,291			
	0 ve 1. grade	788 (61%)	48 (6.1%)		0.243
	2 ve 3. grade	503 (39%)	23 (4.6%)		
RLN anatomy		1,232			0.208
	R1/L1	1,091 (88.5%)	56 (5.1%)		
	R2a/L2a	96 (7.8%)	8 (8.3%)		
	R2b/L2b	38 (3.1%)	4 (10.5%)		
	R3/L3	7 (0.6%)	1 (14.3%)		
RLN trajectory		1,124			0.553
	No feature	950 (84.5%)	56 (5.9%)		
	Fixed	43 (3.8%)	1 (2.3%)		
	Splayed	59 (5.3%)	5 (8.5%)		
	Entrapped	72 (6.4%)	3 (4.2%)		
Weight of thyroid gland lobe (gram)		1,059			0.127
	No VCP	1,008		29.6 ± 32.7 (2–274)	
	VCP	51		32.4 ± 33 (5–204)	
Hyperthyroidism		1,412			
	Yes	1,091 (77.3%)	62 (5.7%)		0.565
	No	321 (32.7%)	21 (6.5%)		
Final diagnosis		1,412			
	Benign	828 (58.6%)	44 (5.3%)		0.148
	Graves disease	149 (10.6%)	14 (9.4%)		
	Malignant	435 (30.8%)	25 (5.7%)		

VCP, vocal cord paralysis; IIONM, intermittent intraoperative nerve monitorization; CIONM, continuous intraoperative nerve monitorization; RLN, recurrent laryngeal nerve; ITA, inferior thyroid artery. *means statistically significant p value.

Temporary VCP rates in operations with the use of IIONM compared with those with the use of CIONM (6.9 vs. 4.2%, *p* = 0.024, respectively), secondary interventions compared to primary interventions (10.8 vs. 5.2%, *p* = 0.033, respectively), RLNs crossing ITA anteriorly, compared to those crossing posteriorly and between the branches (7.9 vs. 3 vs. 3.2%, *p* = 0.000, respectively), extralaryngeal branching nerves compared to the non-branching nerves(7.9 vs. 4.3%, *p* = 0.005, respectively), RLNs entrapped by Berry fibers and/or vascular structure compared to those without entrapment (9.8 vs. 4.1%, *p* = 0.001, respectively) were higher. No significant difference was detected in terms of other factors (Table 2).

**Table 2:** Evaluation of clinical and anatomical factors for temporary VCP by univariant analysis.

**Feature**		***n***	**VCP n (%)**	**OR (95% CI min–max)**	***p***
Age					
	No VCP	1,329		49.2 ± 13.5 (18–89)	0.979
	VCP	78		48.9 ± 11.8 (23–79)	
Gender					
	Female	1,089	61 (5.6%)	0.934 (0.538–1.624)	0.810
	Male	323	17 (5.3%)		
BMI		1,225			0.638
	No VCP	1,170		28.8 ± 6 (16.3–63)	
	VCP	55		28.4 ± 5.4 (18.4–44.4)	
Neck side of the nerve					
	Right	714	43 (6%)	0.824 (0.521–1.304)	0.407
	Left	698	35 (5%)		
Type of nerve monitoring					
	IIONM	692	48 (6.9%)	1.714 (1.073–2.739)	0.024*
	CIONM	720	30 (4.2%)		
Type of intervention					
	Primary	1,329	69 (5.2%)	0.450 (0.216–0.937)	0.033*
	Secondary	83	9 (10.8%)		
Central neck dissection					
	Applied	147	8 (5.4%)	1.018 (0.480–2.160)	0.963
	Not applied	1,265	70 (5.5%)		
RLN-ITA relationship					0.000*
	Anterior to ITA	675	53 (7.9%)	2.578 (0.916–7.255)	0.073
	Posterior to ITA	596	18 (3%)	0.942 (0.313–2.833)	0.915
	Between branches of ITA	125	4 (3.2%)		
Nerve branching		1,412			0.005*
	Non-branching	930	40 (4.3%)	1.904 (1.204–3.012)	
	Branching	482	38 (7.9%)		
Entrapment of RLN by Berry ligament					
	Yes	307	30 (9.8%)	2.528 (1.549–4.124)	0.001*
	No	998	41 (4.1%)		
Zuckerkandl tubercle		1,291			
	0 ve 1. grade	788	43 (5.5%)		0.482
	2 ve 3. grade	503	23 (4.6%)		
RLN anatomy		1,232			0.280
	R1/L1	1,091	53 (4.9%)		
	R2a/L2a	96	6 (6.3%)		
	R2b/L2b	38	4 (10.5%)		
	R3/L3	7	1 (14.3%)		
RLN trajectory		1,124			0.281
	No feature	950	54 (5.7%)		
	Fixed	43	0		
	Splayed	59	4 (6.8%)		
	Entrapped	72	2 (2.8%)		
Weight of thyroid lobe (gram)		1,059			0.052
	No VCP	1,008		29.6 ± 32.8 (2–274)	
	VCP	51		32.8 ± 32.6 (5–204)	
Hyperthyroidism					
	Yes	1,091	57 (5.2%)		0.364
	No	321	21 (6.5%)		
Final diagnosis					
	Benign	828	41 (5%)		0.089
	Graves' disease	149	14 (9.4%)		
	Malignant	435	23 (5.3%)		

VCP, vocal cord paralysis; IIONM, intermittent intraoperative nerve monitorization; CIONM, continuous intraoperative nerve monitorization; RLN, recurrent laryngeal nerve; ITA, inferior thyroid artery. *means statistically significant p value.

Permanent VCP rates in secondary interventions compared to primary interventions (2.4 vs. 0.2%, *p* = 0.009, respectively), and patients with RLNs fixed, splayed, entrapped compared to those having normal RLN courses (2.3 vs. 1.7 vs. 1.4 vs. 0.2%, *p* = 0.043, respectively) were higher. No significant difference was detected in terms of other factors (Table 3).

**Table 3:** Evaluation of clinical and anatomical factors for permanent VCP by univariant analysis.

**Feature**		***n***	**VCP n (%)**	**OR (95% CI min–max)**	***p***
Age		1,412			
	No VCP	1,407		49.2 ± 13.4 (18–89)	0.074
	VCP	5		58.2 ± 6.2 (49–66)	
Gender		1,412			
	Female	1,089	4 (0.4%)	0.841 (0.094–7.549)	1
	Male	323	1 (0.3%)		
BMI		1,230			0.417
	No VCP	1,175		28.8 ± 5.9 (16.3–63)	
	VCP	5		31.5 ± 7.6 (22.9–43.8)	
Neck side of the nerve		1,412			
	Right	714	3 (0.4%)	0.681 (0.113–4.088)	1
	Left	698	2 (0.3%)		
Type of nerve monitoring		1,412			
	IIONM	692	1 (0.1%)	1.714 (1.073–2.739)	0.375
	CIONM	720	4(0.6%)		
Type of intervention		1412			
	Primary	1,329	3 (0.2%)	0.092 (0.015–0.556)	0.009*
	Secondary	83	2 (2.4%)		
Central neck dissection		1,412			
	Applied	147	2 (1.4%)		0.055
	Not applied	1,265	3 (0.2%)	0.172 (0.029–1.040)	
RLN-ITA relationship		1,396			0.351
	Anterior to ITA	675	4 (0.6%)		
	Posterior to ITA	596	1 (0.2%)		
	Between Branches of ITA	125	0		
Nerve branching		1,412			0.504
	Non-branching	930	4 (0.4%)	0.481 (0.054–4.318)	
	Branching	482	1 (0.2%)		
Entrapment of RLN by Berry ligament		1,305			
	Yes	307	0	0.995 (0.991–0.999)	0.597
	No	998	5(0.5%)		
Zuckerkandl tubercle		1,291			
	0 ve 1. grade	788	5 (0.6%)		0.163
	2 ve 3. grade	503	0		
RLN anatomy		1,236			0.062
	R1/L1	1,091	3 (0.3%)		
	R2a/L2a	96	2 (2.1%)		
	R2b/L2b	38	0		
	R3/L3	7	0		
RLN trajectory		1,124			0.043*
	No feature	950	2(0.2%)		
	Fixed	43	1 (2.3%)		
	Splayed	59	1 (1.7%)		
	Entrapped	72	1 (1.4%)		
Weight of thyroid gland lobe (gram)		1,059			0.127
	No VCP	1,008		29.6 ± 32.8 (2–274)	
	VCP	51		32.8 ± 32.6 (5–204)	
**Hyperthyroidism**					
	Yes	1,091	5 (0.5%)		0.594
	No	321	0		
**Final diagnosis**					
	Benign	828	3 (0.4%)		0.716
	Graves disease	149	0		
	Malignant	435	2 (0.5%)		

VCP, vocal cord paralysis; IIONM, intermittent intraoperative nerve monitorization; CIONM, continuous intraoperative nerve monitorization; RLN, recurrent laryngeal nerve; ITA, inferior thyroid artery. *means statistically significant p value.

In the logistic regression analysis, the type of monitoring, type of intervention, entrapment of the nerve in the Berry region, and RLN relationship with inferior thyroid artery were determined as independent risk factors for total VCP (Table 4). The risk of VCP was 2 times higher in those who underwent IIONM compared to those who underwent CIONM, 3.8 times higher in those who underwent secondary intervention compared to those who underwent primary intervention, 2.5 times higher in the entrapment of RLNs in the Berry region compared to those which has no entrapment 2.6 times in patients RLNs crossing ITA anteriorly compared to those crossing posteriorly or between the branches (Table 4).

**Table 4:** Multivariate analysis of risk factors for total VCP with binary logistic regression.

	**Vocal cord paralysis (VCP)**	
	**Odds ratio (95%CI Lower–Upper)**	***p***
**Type of neuromonitoring**		0.008
I-IONM	2.000 (1.195–3.345)	
C-IONM	1 (reference)	
**Type of intervention**		0.001
Primary	0.262 (0.117–0.588)	
Secondary	1 (reference)	
**Berry entrapment**		0.000
Absent	0.406 (0.245–0.6719)	
Present	1 (reference)	
**RLN—ITA relationship**		0.001
Anterior to ITA	2.603 (0.909–7.450)	0.075
Posterior to ITA	0.973 (0.317–2.985)	0.962
Between the branches of ITA	1.00 (reference)	
**Nerve branching**		0.055
Non-branching	0.618 (0.379–1.010)	
Branching	1.00 (reference)	

*Logistic regression OR <1 decreases risk (e.g., VCP risk decreases in RLN posterior to ITA compared to the RLN anterior to ITA), OR > 1 increases risk (e.g., VCP risk increases in branching RLN compared to non-branching RLN)*.

### Mechanisms of RLN Injury

The mechanisms of RLN injury were traction trauma in 57 (68.8%) nerves, thermal injury in 17 (20.5%), unintentional nerve transection in 5 (6%), and mechanical trauma in 3 (3.7%). Rates of mechanisms of RLN injury were significantly different between IIONM and CIONM according to IONM type (*p* = 0.042). Traction trauma was more frequent in IIONM compared to CIONM (80 vs. 53%, respectively), however, mechanical trauma (2 vs. 8.8%, respectively), thermal injury (16 vs. 26.4%, respectively), unintentional transection (2 vs. 11.8%, respectively) were less frequent (Table 5).

**Table 5:** Types of mechanisms leading to RLN injury.

	**Vocal cord paralysis (VCP)**		
	**IIONM VCP**	**CIONM VCP**	***P* = 0.042**
**Traction**	39 (80%)	18 (53%)	
Mechanical trauma	1 (2%)	3 (8.8%)	
Clip or suture	0	0	
**Thermal**	8 (16%)	9 (26.4%)	
Transection	1 (2%)	4 (11.8%)	
Total	49	34	83

*VCP, vocal cord paralysis; IIONM, intermittent intraoperative nerve monitorization; CIONM, continuous intraoperative nerve monitorization; RLN, recurrent laryngeal nerve*.

## Discussion

As far as we know, this study is the most extended study in terms of the number of nerves classified according to the International RLN anatomical classification system.

Despite the positive contribution of IONM to the reduction of RLN paralysis in thyroidectomy, VCP is still among the main post-thyroidectomy complications. In this study, IONM was used in all patients and post-thyroidectomy total VCP rate according to the number of nerves was 5.9%, temporary VCP rate was 5.5%, and permanent VCP was 0.4%. In our results, logistic regression analysis with the formula obtained from the factors that were significant in paired comparison; IIONM, secondary interventions, entrapment of the RLN in the Berry region, RLNs crossing the ITA from the anterior, nerve branching revealed as independent factors increasing the risk of VCP. In our study, the risk of VCP with IIONM was 2 times higher (*p* = 0.008) than with CIONM. The risk of VCP is approximately 3.8 times (*p* = 0.001) higher in secondary interventions compared to primary interventions, and 2.5 times (*p* = 0.000) higher in entrapment of RLN in the Berry region, 2.6 times (*p* = 0.001) higher with the RLNs crossing anterior to ITA compared with those crossing between the branches and posterior to ITA. Although the rate of VCP was 1.6 times higher in branched nerves than in non-branching nerves, the difference was not significant (*p* = 0.055).

Although the temporary VCP rate was lower in CIONM, the permanent VCP rate was higher in CIONM, although the statistical analysis was not significant (0.6 vs. 0.1%, *p* = 0.375).

The prevalence of IONM usage has increased significantly in the last 10 years by both general surgeons and otolaryngologists (10).

The effect of IONM on VCP is still controversial. In some studies, it has been reported that the use of IONM does not have a significant effect on VCP (4, 12). In some meta-analyses regarding this subject, it has been reported that IONM had no significant benefit over visualization in preventing RLN injury, and it should not be considered as standard care and replace visualization (28, 29).

On the other hand, Barczynski et al. demonstrated in their randomized clinical trial that IONM decreased the rate of transient RLN paralysis (30).

Vasileiadai et al. demonstrated in their study that the use of IONM decreased both transient and permanent RLN injuries significantly (31).

Bai and Chen detected that IONM decreased the rates of both temporary and permanent RLN paralyzes in a meta-analysis including 59,380 nerves, and they recommended routine use of IONM, especially in bilateral operations and malignant operations (13).

In our study, the most common cause of VCP was traction trauma (68.8%), similar to other studies (27).

Even though the rate of traction trauma was lower in the CIONM group than IIONM group (53 vs. 80%, respectively), the rates of thermal trauma (16 vs. 26.4%, respectively) and unintentional transection (2 vs. 11.8%, respectively) were higher in the CIONM group. CIONM supports the early detection of signs of traction trauma, contributing to the reduction of VCP associated with traction trauma by changing the action. On the other hand, all of the permanent VCPs in the study emerged after the unintentional transection.

Although CIONM is an effective method for preventing VCPs due to traction trauma, it is unlikely to prevent sudden actions such as transection, cauterization, and clamping (32).

In a study, in which 1,526 patients who were operated on for benign thyroid disease were evaluated, also IIONM and CIONM were compared, the rates of temporary VCP were comparable (2.3 vs. 2.6%, *p* = 0.844, respectively), and it was found that the rate of permanent VCP decreased with CIONM (0.4 vs. 0% *p* = 0.019) (33).

CIONM was also found to be more suitable for evaluating nerve electrophysiology in children (34).

In a comparative study involving 6,029 patients, CIONM was found to be an independent risk reducing factor for both temporary and permanent VCP, and it was found to reduce early VCP by 1.8 times (OR: 0.56) and permanent VCP by 29.4 times (OR: 0.0034) compared to IIONM. Permanent VCP develops in one out of every 4.2 early VCPs with IIONM and in one out of 75 early VCPs with CIONM, the probability of developing permanent VCP after early VCP is 17.9 times lower in CIONM, and it has been demonstrated to be a superior method in preventing VCP (14).

However, the rate of use of CIONM is still very low compared to IIONM (10).

Reoperations for thyroid disease are associated with an increased risk of RLN paralysis (4, 5, 35).

In the retrospective cohort study by Barczynski et al. IIONM significantly reduces the incidence of transient VCP in secondary surgeries compared to only visual identification of nerve (2.6 vs. 6.3%, respectively, *p* = 0.003). Although the difference is not significant, it also decreased the incidence of permanent VCP rate (1.4 vs. 2.4%, respectively, *p* = 0.202) (36).

In this study, secondary intervention is a risk factor for VCP in operations with IONM.

In secondary interventions, the trajectory of RLN changes by 80% and approximately 60% of RLN crosses within the scar tissue. These are important anatomical factors that increase the risk of RLN injury due to difficulties visualizing and identifying the nerve, and our results support this information (36, 37).

Anatomical variations of the RLN are common and these variations may increase the risk of RLN injury due to visual misidentification of the nerve (38).

In the literature, many studies have evaluated the relationship of RLN with other anatomical landmarks (15–20).

A prospective international multicentric study by Liddy et al. was the first study evaluating 1,000 nerves at risk according to the International RLN anatomical classification system.

In the study, nerve trajectory was found to be 77% L1/R1 (normal trajectory), 19.4% L2a/R2a, 3% R2b/L2b, 0.7% R3. In total, 30% of nerves at risk were fixed/splayed/entrapped at the level of the thyroid capsule. The rate of entrapment of the nerve by the ligament of Berry and/or vascular structure in the Berry ligament region was 41%, rate of nerve thinner than 1 mm was found in 16%, and extralaryngeal branching was detected in 28% (22).

In this study, RLN trajectories were evaluated in 1,232 nerves, and it was found to be 88.5% L1/R1 (normal trajectory), 7.8% (L2a/R2a), 3.1% (L2b/R2b), and 0.0% R3. Fixed/splayed/entrapment nerve at the level of thyroid capsule was detected in 179 (15.5%) of 1,124 nerves. Berry entrapment was detected in 307 (23.5%) of 1305 nerves, and nerve branching was detected in 482 (34.1%) of 1,412 nerves.

According to the study of Liddy et al. the rate of normal trajectory was higher, while the rate of lateral or medial acquired abnormal trajectory was lower. In addition, the fixed/splayed/entrapment ratio at the level of the thyroid capsule and the entrapment of the RLN in the Berry ligament region were lower. In the study of Liddy et al. there are 17 centers from 7 geographical regions and 12 countries, and there are centers from our country, too also including our center. However, this difference may be related to differences in postoperative diagnoses and geographical anatomical differences in studies. Anatomical risk factors for RLN injury were evaluated among these anatomical features (22).

For loss of signal (LOS), presence of abnormal RLN trajectory (OR: 2.12, *p* = 0.017), entrapment of RLN in Berry's ligament (OR: 3.25, *p* = 0.007), lateral lymph node dissection (OR: 4.43, *p* = 0.025) for the right side, higher BMI (OR:0.98, *p* = 0.032) were determined as independent risk factors for the left side. However, for both right and left sides, invaded nerve (OR: 18.30, 15.50; *p* = 0.002, *p* = 0.021, respectively), fixed nerve in the thyroid capsule (OR: 16.63, 3.45; *p* = 0.000, *p* = 0.044, respectively), extended nerve dissection (OR: 11.56, 6.42; *p* = 0.000, *p* = 0.008, respectively) were determined as independent risk factors for LOS. For VCP, the fixed nerve in the thyroid capsule (OR: 2.57; *p* = 0.006), an increase in the length of the RLN exposure (OR > 999.99; *p* = 0.006) for the right side, the invaded nerve on both the right (OR: 21.07; *p* < 0.001) and the left side (OR: 43.75; *p* < 0.001) were determined as independent risk factors.

The researchers have recommended that the anatomical and intraoperative characteristics of the RLN, which may affect the risk of nerve injury, may be significantly variable, and the use of IONM should be routine since these cannot be predicted preoperatively (22).

In this study, no significant difference was found in terms of total and transient RLN paralysis, whether there were an abnormal nerve trajectory, clinically important features (fixed/splayed/entrapment) or not. Clinically important features (fixed/splayed/entrapment) were significantly higher in patients with permanent VCP (*p* = 0.043). We believe this is one of the important features that complicate the identification of the nerve. Total (9.8 vs. 4.6%, *p* = 0.001, respectively) and transient (9.8 vs. 4.6%, *p* = 0.001, respectively) VCP were significantly higher in patients with nerve entrapment in the Berry region than in those without. This is an important indicator that the risk of RLN injury is most likely and that traction trauma is the most common in the Berry region and these features are important risk factors.

In this study, extralaryngeal nerve branching (34.1%) was a frequent variation, and the probability of total (8.1 vs. 4.7%, *p* = 0.011, respectively) and transient (7.9 vs. 4.3%, *p* = 0.005, respectively). VCP was significantly higher than for non-branching nerves. In previous studies, it has been revealed that extralaryngeal branching is a risk factor for VCP (39–41).

Similar to our results, the transient VCP rate was found higher in branched nerves than in non-branched ones in a study by Barczynski et al. Increased risk of paralysis has been calculated as 2.98 times more in branched nerves (95 %CI 1.79–4.95; *p* = 0.001). In addition to that, there was no difference in branched and non-branched nerves in terms of permanent VCP rates (1.1 vs. 0.2%, respectively) (41).

In another study by Sancho et al. the VCP rate in branched nerves was higher than in non-branched nerves significantly (15.8 vs. 8.1%, respectively *p* = 0.022), moreover, the probability for VCP was determined 2.2 times higher in branched nerves (95% CI: 1.1–4.5) (40).

Estimated risks for unilateral RLN paralysis were 7.36 times higher for transient paralysis (95% CI: 1.84–29.4; *p* = 0.0061) and 13.25 times higher in permanent paralysis (95% CI: 1.42–123.73; *p* = 0.0204) in branched nerves, compared to non-branching nerves in a study by Casella et al. (39).

In this study, nerve–artery relationship was another anatomical feature evaluated for risk of VCP. In RLNs crossing ITA anteriorly compared to crossing posteriorly or crossing between branches, total VCP (8.4 vs. 3.2 vs. 3.2%, *p* = 0.000, respectively) and transient VCP (7.9 vs. 3 vs. 3.2%, *p* = 0.000, respectively) were significantly higher so that relationship was an independent risk factor for both. There was no significant difference in terms of permanent VCP. We think that a greater traction force is reflected to the nerve in the course of RLN anterior to the ITA by being exposed to more elevation and artificial angulation during the anteromedial traction of the thyroid lobe.

When the nerve is retracted, the maximum tension is reflected in the angulation area and the last 2 cm (42).

RLN-ITA relationship is a potential anatomical feature in terms of RLN paralysis, and we believe it can be evaluated for inclusion in the international RLN anatomy classification.

On the other hand, Sancho et al. had evaluated the anatomical relation of RLN to ITA, they found VCP rates as 15% if the RLN crossed the ITA anteriorly, 14.7% if crossed posteriorly, and 9.1% if crossed between the branches, and they did not determine a significant difference in VCP according to the position of the RLN (*p* = 0.529) (40).

The main limitation of our study is being retrospective. Although our center has standard technical equipment for IIONM, the vagus probe (APS probe) cannot be supplied to every patient. The use of CIONM was preferred in preoperatively predicted high-risk patients and when the vagus probe was accessible. It can be thought that this situation may affect the results related to the effect of IONM.

Although many factors have been evaluated, the presence of thin nerve and wide dissection, which are defined in the international RLN anatomic classification system, were not evaluated, also LOS and dynamic data were not evaluated in the study but only postoperative VCP was evaluated. Since the primary aim of the study was to evaluate RLNs anatomical factors on VCP development, not all dynamic factors were evaluated. However, many of the anatomical variations of the RLN in the international RLN classification system and the RLN–ITA relationship have been evaluated.

In conclusion, anatomic variations of the RLN are common in thyroidectomy. Among the anatomical factors, the RLN–ITA relationship, extralaryngeal branches, and entrapment of the RLN at Berry ligament are important factors affecting the development of postoperative VCP, which may make thyroidectomy high-risk and cannot be predicted preoperatively. Considering the potential anatomical variations of the RLN, IONM can be used in every thyroidectomy, CIONM has more advantages than IIONM, and VCP risk can be reduced with CIONM.

## Data Availability Statement

The original contributions presented in the study are included in the article/supplementary material, further inquiries can be directed to the corresponding author/s.

## Ethics Statement

The studies involving human participants were reviewed and approved by Sisli Hamidiye Etfal Training and Research Hospital. The patients/participants provided their written informed consent to participate in this study.

## Author Contributions

The conception, design, and supervision were contributed by MU and AI. The parts of materials, data collection and/or processing, and literature review were contributed by MK, MTU, and NA in the study. Writing and critical review were done by AI, MU, NA, MK, and MTU. All authors contributed to the article and approved the submitted version.

## Conflict of Interest

The authors declare that the research was conducted in the absence of any commercial or financial relationships that could be construed as a potential conflict of interest.

## Publisher's Note

All claims expressed in this article are solely those of the authors and do not necessarily represent those of their affiliated organizations, or those of the publisher, the editors and the reviewers. Any product that may be evaluated in this article, or claim that may be made by its manufacturer, is not guaranteed or endorsed by the publisher.
